# An Orf-Virus (ORFV)-Based Vector Expressing a Consensus H1 Hemagglutinin Provides Protection against Diverse Swine Influenza Viruses

**DOI:** 10.3390/v15040994

**Published:** 2023-04-18

**Authors:** Gabriela Mansano do Nascimento, Dina Bugybayeva, Veerupaxagouda Patil, Jennifer Schrock, Ganesh Yadagiri, Gourapura J. Renukaradhya, Diego G. Diel

**Affiliations:** 1 Department of Population Medicine and Diagnostic Sciences, Animal Health Diagnostic Center, College of Veterinary Medicine, Cornell University, Ithaca, NY 14850, USA; 2 Department of Animal Sciences, Center for Food Animal Health, College of Food, Agricultural, and Environmental Sciences, The Ohio State University, Wooster, OH 44691, USA

**Keywords:** swine influenza, vaccines, H1, vectored vaccines, Orf virus

## Abstract

Influenza A viruses (IAV-S) belonging to the H1 subtype are endemic in swine worldwide. Antigenic drift and antigenic shift lead to a substantial antigenic diversity in circulating IAV-S strains. As a result, the most commonly used vaccines based on whole inactivated viruses (WIVs) provide low protection against divergent H1 strains due to the mismatch between the vaccine virus strain and the circulating one. Here, a consensus coding sequence of the full-length of HA from H1 subtype was generated in silico after alignment of the sequences from IAV-S isolates obtained from public databases and was delivered to pigs using the Orf virus (ORFV) vector platform. The immunogenicity and protective efficacy of the resulting ORFV^Δ121^conH1 recombinant virus were evaluated against divergent IAV-S strains in piglets. Virus shedding after intranasal/intratracheal challenge with two IAV-S strains was assessed by real-time RT-PCR and virus titration. Viral genome copies and infectious virus load were reduced in nasal secretions of immunized animals. Flow cytometry analysis showed that the frequency of T helper/memory cells, as well as cytotoxic T lymphocytes (CTLs), were significantly higher in the peripheral blood mononuclear cells (PBMCs) of the vaccinated groups compared to unvaccinated animals when they were challenged with a pandemic strain of IAV H1N1 (CA/09). Interestingly, the percentage of T cells was higher in the bronchoalveolar lavage of vaccinated animals in relation to unvaccinated animals in the groups challenged with a H1N1 from the gamma clade (OH/07). In summary, delivery of the consensus HA from the H1 IAV-S subtype by the parapoxvirus ORFV vector decreased shedding of infectious virus and viral load of IAV-S in nasal secretions and induced cellular protective immunity against divergent influenza viruses in swine.

## 1. Introduction

Influenza A viruses have been circulating in swine worldwide. Currently the three enzootic subtypes of influenza A viruses circulating in pigs are H1N1, H1N2, and H3N2 [1]. The swine influenza virus (IAV-S) evolutionary history reflects multiple introductions of influenza viruses into pigs from other species, mainly from avian species and humans [2,3,4,5,6,7]. The H1N1 subtype, known as classical H1N1 virus (cH1N1), was first isolated from pigs in 1930 and predominated in North America until 1998, when a reassorted H3N2 emerged and became widespread [5,8,9]. Swine influenza is one of the major causes of acute respiratory disease outbreaks in pigs [10,11]. Moreover, IAV-S has been recognized as a public health threat since its original isolation from a human in 1974 [12]. The outbreak of a pandemic H1N1 (pdmH1N1) virus in humans in 2009, a triple reassortant H1N1 virus presenting gene segments from swine, human, and avian influenza viruses, highlighted the human-swine interface of influenza demonstrating that swine populations play a crucial role in the emergence and interspecies transmission of IAV-S [13,14,15]. Similarly, a variant H3N2, a swine-origin influenza A virus (IAV-S), was recently detected in humans, predominantly children, who attended agricultural fairs in the USA [16]. In addition, recent IAV-S surveillance in China showed high seropositivity for a Eurasian avian-like (EA) H1N1 virus in swine workers [17].

The two major contributors to the genetic diversity of IAV-S are the high rate of mutation of the *Orthomyxoviridae* family members and the reassortment source that swine represent for these viruses. The first is intrinsically related to the RNA genome of orthomyxoviruses and the inherent error-prone characteristic of the viral RNA polymerase [18]. This process, known as antigenic drift, plays an important evolutionary role whereby IAV-S can evade the immune system, mainly by a continuous variation in the most immunogenic sites of HA [19]. The second takes advantage of the existence of two types of sialic acid receptors in swine: α(2,3) and α(2,6) linkages [20]. This makes swine susceptible to IAVs of mammalian and avian origin. These receptors are used by the IAV-S hemagglutinin protein (HA) to bind host cells. IAV-S and other mammalian-adapted IAVs tend to preferentially bind to sialic acid receptors that have α(2,6) linkages. In contrast, most avian influenza viruses have a binding preference for α(2,3) linked sialic acids [21,22]. When two or more IAVs coinfect the same swine cell, viral genome segments can reassort, leading to the emergence of novel viruses with pandemic potential [22,23].

Vaccination is the most common strategy to control influenza in swine, but due to the substantial antigenic diversity of circulating strains in different parts of the world, conventional approaches to develop efficient vaccines have achieved limited success. One way to minimize the sequence diversity between vaccine strains and circulating viruses is to create a computationally designed sequence based on contemporary virus isolates to decrease the genetic and antigenic distances of the vaccine and field viruses [24]. Currently, most of the licensed IAV-S vaccines in the United States are based on whole inactivated viruses (WIVs) of the H1 and H3 subtypes. Additionally, a modified live vaccine (MLV) and a RNA vaccine are also approved by United States Department of Agriculture (USDA) [25,26]. Overall, the WIV vaccines are effective in providing protection against homologous viruses, but not against heterologous strains [27]. Killed-virus vaccines induce mainly humoral immunity, while broad-protection also requires cell-mediated immune responses [28]. On the other hand, modified MLV vaccines stimulate both humoral and cellular arms of the immune response, providing better levels of heterosubtypic immunity and cross-protection [28,29,30]. However, the potential for reassortment between field strains and the vaccine virus is a major safety concern regarding the use of live-virus vaccines [31]. Currently, there is just one RNA-based vaccine to use in swine licensed by USDA that demonstrated similar levels of protection as a licensed WIV [32]. Notably, while human influenza vaccines are updated every year to ensure the closest match with circulating strains, swine influenza vaccines are outdated and contain older strains that are no longer circulating in pigs [33].

Here we selected Orf virus (ORFV) as a viral vector to deliver a consensus H1 IAV-S antigen in swine, based on its inherent ability to modulate the immune system and induce protective responses against viral agents in swine [34,35,36,37,38]. This parapoxvirus is a member of the *Poxviridae* family with a large 138kb double-stranded DNA genome containing 131 open reading frames (ORFs). ORFV encodes several immunomodulatory proteins (IMPs), including ORF 121 (ORFV121), a nuclear factor-kappa beta (NF-κB) signaling pathway inhibitor, which is an important virulence determinant of ORFV that is non-essential for virus replication in vitro [39]. ORFV represents a promising vector platform to deliver foreign antigens in animals due to its narrow natural host range (sheep and goats), self-limiting infection, lack of neutralizing antibodies against the vector, and natural immunogenicity, which induces humoral and T cell responses against heterologous antigens, and has been successfully used to deliver antigens in livestock species [40,41]. Previously, our group demonstrated that an ORFV vector based on the strain IA82 (OV-IA82) elicits protective levels of neutralizing antibodies against rabies virus (RABV) in pigs and cattle [40], and against porcine epidemic diarrhea virus (PEDV) in swine [36,42]. More importantly, recently, we demonstrated that an ORFV-vector expressing IAV-S HA or HA and NP proteins induced high levels of antibodies and T cell responses to IAV-S, thus, providing protection against a homologous challenge and proving that the versatile ORFV platform is suitable to control IAV-S in swine [37].

Given the diversity of IAV-S, there is a need for novel/alternative vaccine platforms to improve and broaden the degree of protection against IAV-S. New approaches are essential not only to protect swine, but also to mitigate the risk of potential transmission and infection in humans. Here, we showed that the delivery of the consensus HA from H1 IAV-S subtype by the ORFV vector induced protection against divergent influenza viruses in swine.

## 2. Materials and Methods

### 2.1. Cells and Viruses

Primary ovine fetal turbinate (OFTu), swine turbinate cells (STU), and Madin–Darby Canine Kidney (MDCK) cells) cells were cultured at 37 °C with 5% CO^2^ in minimum essential medium (MEM) or Dulbeco’s modified Eagle’s medium (DMEM) supplemented with 10% FBS, 2 mM L-glutamine, and containing penicillin (100 U mL^−1^), streptomycin (100 µg mL^−1^), and gentamicin (50 µg mL^−1^). ORFV strain IA82 (OV-IA82) (provided by Dr. Daniel Rock at the University of Illinois at Urbana-Champaign) [39,41,42,43] was used as the parental virus to construct the recombinant ORFV expressing the influenza A virus of swine (IAV-S) consensus H1 glycoprotein (HA). Swine influenza virus H1N1 A/Swine/OH/24366/2007 (OH/07, provided by Dr. Gourapura’s lab at OSU), a H1N1 virus, belonging to clade gamma, and pandemic H1N1 A/California/04/2009 (CA/09, obtained from the National Veterinary Services Laboratory [NVSL], Ames, Iowa), belonging to the clade “new pandemic” (npdm), were used for virus challenge and as coating antigen in whole-virus ELISAs. Both H1N1 viruses were propagated in MDCK cells using DMEM containing TPCK-treated trypsin (2 µg mL^−1^) and 25 mM HEPES buffer. All the IAV-S isolates used in cross-reactivity assays were obtained from NVSL. These isolates included A/Swine/Iowa/A02424852/2020 (Clade gamma), A/Swine/South Dakota/A02156993/2018 (Clade gamma-2-beta-like), A/Swine/Missouri/A02479312/2020 (Clade npdm), A/Swine/Michigan/A02524810/2020 (Clade npdm), A/Swine/Texas/A02245632/2020 (Clade beta), A/Swine/Oklahoma/A02245707/2020 (Clade beta), A/Swine/Minnesota/A01785306/2017 (Clade alpha), A/Swine/Iowa/A02479151/2020 (Clade delta 1), A/Swine/Oklahoma/A02214419/2017 (Clade delta 1), and A/Swine/South Dakota/A02524887/2020 (Clade gamma). More information regarding these isolates is presented in Table 1.

### 2.2. Construction of Recombinant Plasmids

The consensus full-length coding sequence of HA from the H1 subtype (conH1) was generated in silico after analysis and alignment of a final set of 63 sequences of IAV-S isolated between 2014–2017 from the H1 subtype obtained from public databases (Table S1). Restriction endonuclease sites required for insertion into the ORFV121 locus of the ORFV genome and early poxviral transcription termination signals (TTTTTNT) were removed from the conH1 sequence through silent nucleotide substitutions. The FLAG tag epitope coding sequence was added to the 5′ of the HA consensus coding sequence under the control of the vaccinia virus late I1L promoter [44]. Finally, the coding sequences of the restriction sites for HindIII and SalI were added to the 5′ and 3′ ends of the conH1. The DNA fragment containing the full-length of the conH1 coding sequence under the control of the I1L promoter and FLAG tag epitope was chemically synthesized (GenScript^®^, Piscataway, NJ, USA) and subcloned into the poxviral pUC57-ORFVΔ121-loxP-EGFP transfer vector, as previously described [37]. Correct cloning of conH1 was confirmed by restriction enzyme analysis in 1% agarose gel.

### 2.3. Generation and Characterization of the Recombinant Viruses

The full-length conH1 sequences was inserted into the ORFV121 locus [42,43] of the ORFV genome by homologous recombination between the parental ORFV strain IA82 and the recombination cassette pUC57-ORFVΔ121-loxP-EGFP, as previously described [36,37]. The presence of conH1 and absence of the ORFV121 sequence in purified recombinant virus were confirmed by PCR screening. Two pairs of internal primers were used for the PCR amplification of conH1, namely IAV-S-conH1-Fw-5′-ACTGCAAGCTTTATTTAAAAGTTGTTTGGTGAACTTAAATGGACTACAAAGACGATGAGACAAGAAAG-3′ and IAV-S-conH1-Rv-5′-GAGGTGTCGACTTAAATACATATTCTACACTGTAAAGAC-3′ (1774 bp), and for the amplification of ORFV121 (401 bp), ORF121-int-Fw-5′- CCTCGGAAAAGAGCAGACAC-3′ and ORF121-int-Rv-5′-CTTCATCAGGCAGTCGTTCA-3′. PCR amplicon analysis followed electrophoresis in 1% agarose gel. Insertion and integrity of full-length conH1 and the identity and integrity of ORFVΔ121 were further [37] confirmed by sequencing on the Illumina Mi-Seq sequencing platform (Illumina, San Diego, CA, USA) using a Nextera XT DNA library preparation kit (Illumina, San Diego, CA, USA).

### 2.4. Expression of conH1 by the ORFV^Δ121^conH1 Recombinant Virus In Vitro

Expression of conH1 by the recombinant virus was assessed by indirect immunofluorescence assay (IFA), flow cytometry (FC), and Western blot (WB), as previously described [37]. Briefly, for the IFA and FC, expression of the heterologous protein in vitro was assessed in permeabilized and non-permeabilized OFTu cells infected with the ORFV^Δ121^conH1 recombinant virus by using an anti-FLAG tag epitope monoclonal mouse antibody (GenScript^®^, Piscataway, NJ, USA).

For the IFA, the goat anti-mouse IgG monoclonal antibody DyLight^®^ 594 (Bethyl Laboratories Inc., Montgomery, TX, USA) was used at 1:300 as the secondary conjugated-antibody. All antibody dilutions were prepared in PBS with 1% bovine serum albumin (BSA), and the cells were visualized under a fluorescence microscope.

For the FC, the antibodies were diluted in Perm/Wash 1× solution for use in permeabilized cells, or in FACS buffer for non-permeabilized cells. The negative control well only received FACS buffer without any antibody. After 30 min of primary antibody incubation, cells were washed and probed with Alexa fluor 594 goat anti-mouse antibody (Invitrogen, Carlsbad, CA, USA) at 1:300. Following the 30 min incubation, the cells were washed, resuspended with 150 µL of PBS 1×, and stored in the dark at 4 °C until data acquisition with an Attune NxT Flow Cytometer. Gates were adjusted based on the mock-infected cells. Gating and data analyses were performed using the FlowJo software (FlowJo V10, Becton, Dickinson & Company; BD, Franklin Lakes, NJ, USA).

Expression of conH1 by the ORFV^Δ121^conH1 recombinant virus was also assessed by Western blot. Briefly, OFTu cells were cultured in 6-well plates and were infected with a high MOI of ORFV^Δ121^conH1 recombinant virus (MOI = 10) and harvested at 12, 24, 48, and 72 hpi. Non-infected OFTu cells were used as the negative control. Cells were lysed, and Western blot was performed as previously described [40]. The constitutively expressed protein β-actin was chosen as the loading control. The membrane was incubated with a monoclonal antibody against β-actin (Santa Cruz Biotechnology Inc., Santa Cruz, CA, USA) at 1:1000 for 2 h at 37 °C. After washing, the membrane was incubated with IRDye 800CW-labeled secondary antibody (LI-COR Biosciences, Lincoln, NE, USA) for 1 h at 37 °C, washed, and developed by the ChemiDoc MP Imaging System (Bio-Rad, Hercules, CA, USA).

### 2.5. Stability of the ORFV^Δ121^conH1 Recombinant Virus in Cell Culture

The stability of the conH1 gene inserted into the ORFV121 locus of the ORFV^Δ121^conH1 genome was evaluated after serial passages (10 passages) of the recombinant virus at 1 MOI in OFTu cells. After 10 passages, a 24-well plate containing OFTu cells was infected with approximately 1 MOI of the ORFV^Δ121^conH1 recombinant virus at passages 1, 5, or 10 and incubated for 24 hpi. Cells were then fixed and permeabilized and stained as described above. A the goat anti-mouse IgG antibody DyLight^®^ 488 conjugate (Bethyl Laboratories Inc., Montgomery, TX, USA) was used as secondary detection antibody.

### 2.6. Cross-Reactivity of ORFV^Δ121^conH1 against Divergent Porcine H1N1 Antisera

The cross-reactivity between the ORFV^Δ121^conH1 recombinant virus and serum of pigs infected with 3 different strains of H1N1 IAV-S was assessed in vitro. OFTu cells were plated in a 24-well plate and infected with ORFV^Δ121^conH1 recombinant virus at 0.1 MOI for 48 hpi. After incubation, the cells were fixed, permeabilized with 0.2% Triton X-100-PBS, and incubated with serial dilutions of the H1N1 antisera developed against A/Sw/CA/04/2009 (clade npdm), A/Sw/IL/00685/2005 (clade delta 2), or A/Sw/KY/02086/2008 (clade beta) strains. These antisera presented hemagglutination inhibition (HI) titers of 1:80, 1:160, and 1:80 against the homologous viruses, respectively. After 1 h incubation, the plate was washed with PBS (3 times) and incubated with an anti-swine IgG secondary antibody DyLight^®^ 488 conjugate (Bethyl Laboratories Inc., Montgomery, TX, USA) to be further visualized under a fluorescence microscope.

### 2.7. Replication Kinetics

Replication properties of ORFV^Δ121^conH1 recombinant virus were assessed in vitro in OFTu and primary swine turbinate (STu) cells cultured in 12-well plates. Multistep (MOI = 0.1) and single step (MOI = 10) growth curves were performed in OFTu cells. Cells were collected at 6, 12, 24, 48, and 72 hpi. Uninfected OFTu and STu were used as controls (0 hpi). Virus titers were determined on each time point using Spearman and Karber’s method [45] and expressed as tissue culture infectious dose 50 (TCID_50_) per milliliter.

### 2.8. Assessment of Protection against Divergent H1N1 Swine Influenza Viruses after Vaccination with ORFV^Δ121^conH1 in Piglets

#### 2.8.1. Animal Immunization-Challenge Experiment

The immunogenicity and protective efficacy of the ORFV^Δ121^conH1 recombinant virus against divergent strains were assessed in pigs. The viruses used for the challenge were A/Sw/OH/24366/2007 (H1N1) (OH/07) [46] and A/California/04/2009 (H1N1) (CA/09) strain. Forty-one 3-week-old specific pathogen free pigs, seronegative for IAV, were randomly allocated to five experimental groups as follows: Group 1, sham-immunized/mock-challenged *(n* = 6); Group 2, ORFV^Δ121^conH1-immunized/OH/07-challenged (*n* = 9); Group 3, ORFV^Δ121^conH1-immunized/CA/09-challenged (*n* = 9), Group 4, sham-immunized/CA/09-challenged (*n* = 9); Group 5, sham-immunized/OH/07-challenged (*n* = 8) (Table 2).

All immunizations were performed via the intramuscular (IM) route by injection of 2 mL of a virus suspension containing 10^7.5^ TCID_50_ mL^−1^ or 2 mL of MEM. Animals from Groups 1, 4, and 5 were sham-immunized with MEM. Animals from Groups 2 and 3 were immunized with ORFV^Δ121^conH1. All animals were immunized on day 0 and received a booster immunization on day 21 post-vaccination (DPV). The pigs from Groups 2 and 5 were challenged with a virus suspension of the OH/07 virus intranasally and intratracheally (1 × 10^7^ TCID_50_ mL^−1^/route) on 40 DPV. Similarly, the pigs from Groups 3 and 4 were challenged with a virus suspension of the CA/09 virus (1 × 10^7^ TCID_50_ mL^−1^/route) intranasally and intratracheally on 40 DPV [47].

Animals were monitored daily. Serum samples and nasal swabs were collected on days 0 and 21 post-vaccination (DPV0 and DPV21) and days 0, 2, 4, and 6 post-challenge (DPC0, DPC2, DPC4, and DPC6). All animals were handled in accordance with the Animal Welfare Act and the animal immunization challenge studies were conducted at The Ohio State University (OSU), following the guidelines and protocols approved by the Institutional Animal Care and Use Committee (IACUC approval no. 2017R00000016).

#### 2.8.2. Humoral Responses against Divergent IAV-S Viruses

IAV-S-specific IgG immune responses elicited by immunization with ORFV^Δ121^conH1 were assessed by whole-virus ELISA of serum and bronchoalveolar lavage, respectively. A panel of 12 WIV antigens for IgG-ELISA were prepared as described previously [37], with some modifications. Briefly, ultra-centrifugation on a 30% sucrose cushion of virus culture supernatant was performed using an Optima-L 100K ultracentrifuge (Beckman Coulter, Brea, CA, USA) at 17,000 RPM for 1.5 h. The virus pellet was resuspended in DMEM, and heat inactivation of the virus was carried out using a water bath at 56 °C for 30 min. Determination of the optimal coating antigen concentration and dilution of secondary antibodies were performed by checkerboard titration.

To detect IAV-S specific total IgG, Immulon 1B ELISA plates (Thermo Fisher Scientific, Waltham, MA, USA) were coated with 250 ng/well in 100 μL volume of a concentrated and heat-inactivated panel of IAV-S virus in bicarbonate/carbonate coating buffer (15 mM sodium carbonate, 35 mM sodium bicarbonate, pH 9.6) in duplicate wells. After 1.5 h incubation at 37 °C, plates were washed 3 times with PBS-T (1X PBS with 0.5% Tween-20) and blocked overnight with 200 μL/well of blocking solution (5% milk in PBS-T) at 4 °C. Blocking reagent was removed and plates washed 3 times with PBS-T. Test and control serum samples were diluted (1:50) in PBS-T 5% non-fat dry milk, and 100 µL of diluted samples were added to paired coated and uncoated control wells and incubated at RT for 1 h. Unbound antibodies were washed with PBS-T (3 times) and plates were incubated with biotinylated secondary antibodies against swine IgG (Bethyl Laboratories Inc., Montgomery, TX, USA) diluted in blocking buffer (1:4000) for 1 h at RT, followed by washing (3 times) and incubation with streptavidin–HRP conjugate (Pierce, Rockford, IL, USA) diluted in blocking solution (1:4000) for 1 h at RT. Streptavidin–HRP conjugate reactions were developed with 3,3′,5,5′-tetramethylbenzidine substrate (TMB) (KPL, Gaithersburg, MA, USA). Finally, the colorimetric reaction was stopped by adding 100 μL 1N HCl solution per well. Optical density (OD) values were measured at 450 nm using a microplate reader (Tecan, Mannedorf, Switzerland). OD values for each test and control samples were normalized to the OD value of uncoated wells. All assay formats were pre-optimized using serum samples from animals of known serological status.

#### 2.8.3. Virus Titration

Infectious IAV-S titers in the swabs and tissue samples were determined by Spearman and Karber’s method and expressed as TCID_50_ mL^−1^. Briefly, 10-fold serial dilutions of the samples were transferred to a 96-well plate pre-seeded with MDCK cells 24 h earlier and incubated at 37 °C for 1 h. After 1 h of adsorption, the inoculum was removed and fresh DMEM containing 2 μg mL^−1^ of TPCK-treated trypsin was added to the cells. After 48 h of incubation at 37 °C, cells were fixed with 3.7% formaldehyde, washed 3 times with PBS and permeabilized with 0.2% PBS-Triton X-100 for 10 min at RT. Virus-positive MDCK cells were detected by immunofluorescence assay using 1:500 of a mouse monoclonal antibody (mAb) targeting the conserved nucleoprotein (NP) of influenza virus (IAV-NP HB-65 462 mAb; kindly provided by Drs. Eric Nelson and Steve Lawson at SDSU), followed by incubation with goat anti-mouse IgG monoclonal antibody DyLight^®^ 488 (Bethyl Laboratories Inc., Montgomery, TX, USA) at 1:350 as described in “Immunofluorescence” from this section. The viral titer was defined as the reciprocal of the highest dilution of the virus where there was infection/replication as evidenced by the presence of fluorescent foci. Appropriate positive and negative control samples were included for all the plates.

#### 2.8.4. Real-Time Reverse Transcriptase PCR (rRT-PCR)

Virus shedding in nasal secretions and viral load in lungs were evaluated by rRT-PCR. Viral nucleic acid was extracted from the nasal swabs and lung tissue homogenates. The lung tissue from the euthanized pigs was collected and lung lysates were prepared in DMEM without serum. Approximately 2–5 g of lung tissue of individual pigs was minced and homogenized for 2 min in a Stomacher 400 laboratory blender (Seward, Long Island, NY), clarified by centrifugation, and the supernatant was collected, aliquoted, and frozen at −80 °C until used [37,48]. All RNA extractions were performed at using the MagMax Core extraction kit (Thermo Fisher, Waltham, MA, USA) and the automated KingFisher Flex nucleic acid extractor (Thermo Fisher, Waltham, MA, USA) following the manufacturer’s recommendations. The presence of IAV-S RNA was assessed using an RNA-to-Ct one-step kit (Applied Biosystems, Waltham, MA, USA) and custom designed primers and probe (Prime Time qPCR probe assays, Integrated DNA Technologies, USA) targeting the conserved NP gene [37]. Amplification and detection were performed using the CFX96 Touch Real-time PCR Detection System (Bio-Rad Laboratories, Hercules, CA, USA), under the following conditions: 10 min at 48 °C for reverse transcription, 10 min at 95 °C for polymerase activation, 40 cycles of 15 s at 95 °C for denaturation, and 60 s at 60 °C or annealing and extension. A standard curve was established by using 10-fold serial dilutions from 10^−1^ to 10^−8^ of either OH07 or CA09 virus suspension containing 10^5.25^ TCID_50_ mL^−1^ and 10^5.38^ TCID_50_ mL^−1^, respectively. Relative viral genome copy numbers were calculated based on the standard curve derived from a four-parameter logistic regression analysis and determined using the CFX Maestro software (Bio-Rad Laboratories, Hercules, CA, USA) as log10 genome copy numbers mL^−1^. Positive and negative amplification controls were run side by side with test samples.

#### 2.8.5. Cellular Immune Responses

On the day of the necropsy (DPC6), heparinized blood was collected for isolation of peripheral blood mononuclear cells (PBMCs). We also collected bronchoalveolar lavage (BAL) and tracheobronchial lymph nodes (TBLN) tissue for isolation of mononuclear cells (MNCs). The frequency of T cell subsets secreting IFN-γ and IL-17A following recall stimulation with OH/07 or CA/09 viruses, depending on the challenge virus used for group, was measured using intracellular cytokine staining (ICS) flow cytometry assay, as previously described [49,50,51]. Briefly, 5 million cells were seeded per well in a 48-well flat bottom plate in 1 mL of culture medium (RPMI 1640 10% FBS) in the presence of recombinant porcine IL-2 and OH07 or CA09 (0.1 multiplicity of infection-MOI) for 48 h (hrs) in vitro. For the last 6 h of the incubation period, protein transport inhibitor Brefeldin A (GolgiPlug) was added. At the end of incubation, cells were harvested, washed, blocked with 1% normal rabbit serum, and separated into an appropriate number of wells in a 96-well round bottom plate for surface and intracellular cytokine labeling. Appropriate isotype control antibodies were included as negative controls. For FACS panels with purified/unlabeled monoclonal antibody (mAb), the cells were first labeled with purified mAb and its corresponding secondary antibody followed by blocking with 1% normal mouse serum. This step was followed by labeling with other cell markers together as a cocktail. Cells were transferred to a 96-well round bottom plate, washed twice in 200 µL FACS buffer/well, and subjected to surface labeling using fluorochrome-conjugated mAbs against indicated markers and their corresponding isotype controls at pre-titrated concentrations in 50 µL of FACS buffer for 30 min at 4 °C. Cells were then fixed using 1% paraformaldehyde at 4 °C for 30 min and resuspended in 200 µL FACS buffer.

For intracellular labeling, cells were washed once and permeabilized with 1% saponin for 45 min at room temperature. Subsequently, cells were washed with saponin wash buffer (0.1% saponin) and incubated with fluorochrome conjugated mAbs against indicated markers and their corresponding isotype controls using pre-titrated concentrations in 50 µL final volume of saponin wash buffer containing 1% normal rabbit serum for 45 min at 4 °C. Cells were washed once in saponin wash buffer and labeled with the indicated secondary antibodies for 45 min at 4 °C. Cells were washed once and resuspended in 200 µL FACS buffer and then transferred to FACS tubes and analyzed using a live cell gate in a BD FACS Aria II flow cytometer. For each sample, 100,000 events were acquired. The data were analyzed using FlowJo software (FlowJo V10, BD Biosciences, Franklin Lakes, NJ, USA) and plotted using Prism version 9 (GraphPad Software, San Diego, CA, USA).

Cells were immunostained for T helper/memory cells (CD3+CD4+CD8α+β−) and cytotoxic T lymphocytes (CTLs) (CD3+CD4+CD8α+β+) using specific immune markers, including purified or fluorochrome labeled antibodies anti-porcine CD3 (Southern biotech, Birmingham, AL, USA), CD4α (Southern biotech, Birmingham, AL, USA), CD8α (Southern biotech, Birmingham, AL, USA), CD8β chain (BD Biosicences, Franklin Lakes, NJ, USA), IFN-γ and IL-17A, and their corresponding isotype controls at previously titrated and optimized concentrations. For positive and negative populations, quadrant markers were set, and these were controlled by non-stained samples and samples incubated with only isotype control antibodies. Lymphocyte subpopulations were separated initially by CD3+ and CD3− gates. The frequency of each individual type of lymphocyte was expressed as the frequency (percentage) of these cells within the 100,000 cells counted.

#### 2.8.6. Statistical Analysis

Statistical analysis was conducted. The data’s normality was tested using the Shapiro–Wilk test, followed by mean comparison. Means between more than two groups was carried out using two-way ANOVA for normal data or nonparametric analog of one-way ANOVA with Kruskal–Wallis or Mann–Whitney U tests for non-normal data, and one-way ANOVA with the Tukey multiple comparison post-test for pairwise comparison. Comparison of means between two groups was performed using an unpaired *t*-test for normal data or the Mann–Whitney test for non-normal data. Significance was determined by a *p* value of less than 0.05. Flow cytometry data was analyzed using Flow Jo software (FlowJo V10, BD Biosciences, Franklin Lakes, NJ, USA).

## 3. Results

### 3.1. Generation and Characterization of the ORFV^Δ121^conH1recombinant Virus

**Figure 1:**
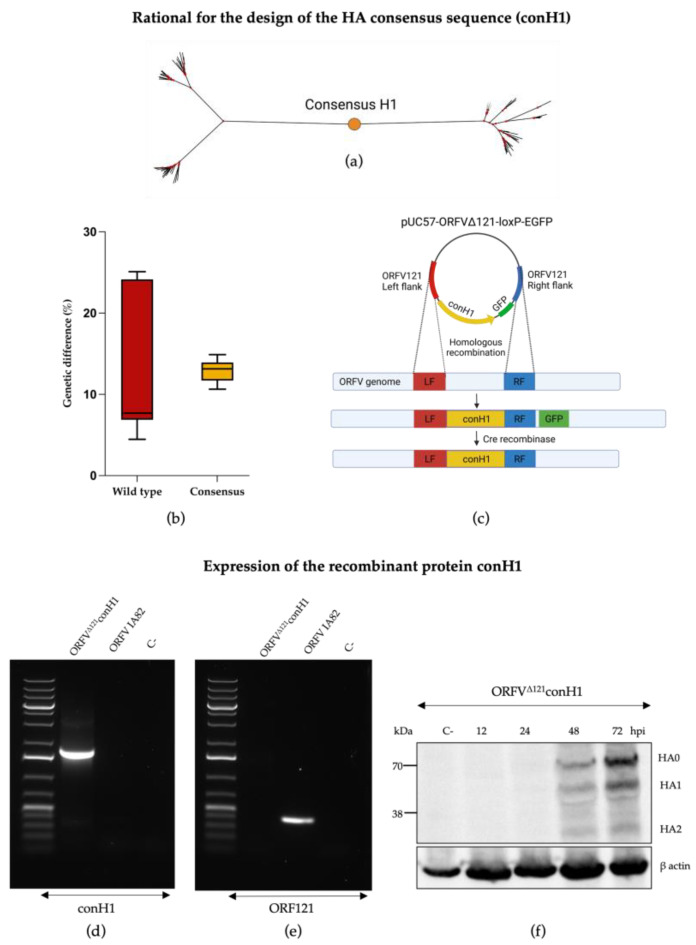
Generation of recombinant ORFV^Δ121^conH1. (**a**) Phylogenetic tree representing the placement of conH1 construct (red dot) relative to the HA sequences used for its design. (**b**) The boxplots show the genetic distance between the circulating strains used to design the consensus HA sequence (in red) and how the consensus-based HA (in yellow) for the H1 subtype was able to reduce this distance. (**c**) Schematic representation of the pUC57-ORFV^Δ121^conH1-loxP-EGFP transfer plasmid and the ORFV genome depicting the ORFV121 insertion site and flanking regions (left flanking—LF, right flank—RF) used to generate the recombinant ORFV^Δ121^conH1. Following homologous recombination, the resulting virus genome contained the insertion of the consensus H1 and the GFP reporter gene into the ORFV121 gene locus. After virus selection, Cre recombinase was used to remove the GFP from the 121 locus, resulting in the final recombinant ORFV^Δ121^conH1 depicting exclusively the consensus H1 sequence into the ORFV121 gene locus. (**d**) Agarose gel demonstrating PCR amplification of ORF121 gene sequences (401 bp) on the wild-type virus and its absence on recombinant ORFV^Δ121^conH1. (**e**) Agarose gel demonstrating PCR amplification of conH1 gene (1774 bp) from the ORFV^Δ121^conH1 virus and lack of detection in the wild-type virus genome. The negative control on the picture comes from the respective PCR reaction using MiliQ water instead of DNA. (**f**) Western blot assay demonstrating expression of conH1 (~70 kDa) by the recombinant ORFV^Δ121^conH1 in OFTu cells infected at MOI = 5 and harvested at 12, 24, 48, and 72 hpi culture in vitro. Cell lysates from non-infected OFTu cells were used as negative controls. Blot was developed with a FLAG tag epitope-specific mAb (Figure 1c was adapted from “Custom Plasmid Maps 1” with permission from BioRender.com (2023). Retrieved from https://app.biorender.com/biorender-templates [52]).

Phylogenetic analyses based on the nucleotide sequence display the designed conH1 in the center of the phylogenetic tree (Figure 1a). The genetic distance between conH1 was dramatically reduced in comparison with the HA sequences from circulating strains from H1 subtype used for the in silico design of conH1 (Figure 1b). The full-length consensus H1 protein of IAV-S was inserted into gene locus 121 [43] of the ORFV genome (ORFV121) by homologous recombination (Figure 1c). After deletion and purification, deleted ORFV121 gene sequences were not detected in the purified recombinant virus (Figure 1d), while conH1 sequences were detected in the recombinant virus but not in the wild-type ORFV genome (Figure 1e). The presence of conH1 and efficient processing and cleavage of conH1 HA0 into HA1 and HA2 subunits were detected in ORFV^Δ121^conH1-infected cells by Western blot (WB) assay (Figure 1f). The complete genome sequence of the ORFV^Δ121^conH1 recombinant virus confirmed the insertion of the full-length conH1 sequence of IAV-S, the integrity of ORFV genome, and the complete deletion of the virulence determinant ORFV121.

**Figure 2:**
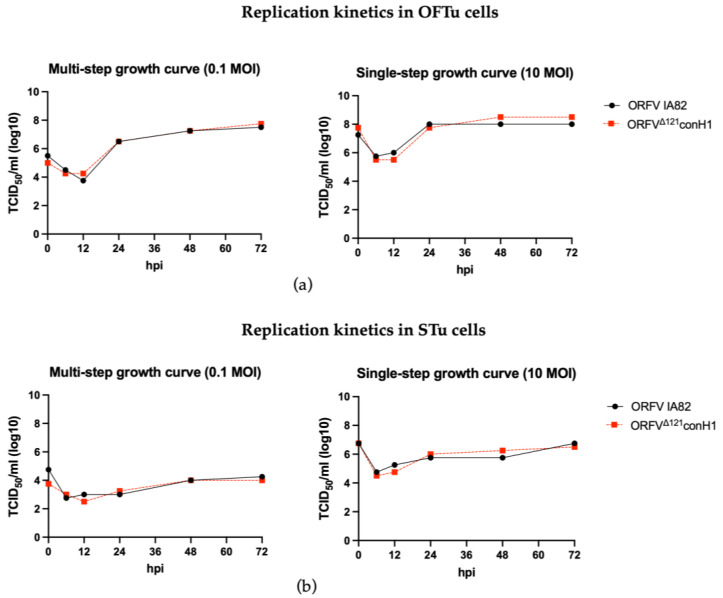
Replication kinetics of the recombinant ORFV^Δ121^conH1. (**a**) Multi- (**left**) and single-step growth curves (**right**) of the recombinant ORFV^Δ121^conH1 were compared to the wild-type virus in primary OFTu cells. (**b**) Multi- (**left**) and single-step growth curves (**right**) of the recombinant ORFV^Δ121^conH1 were also performed in primary STu cells. The virus titers were determined by Spearman and Karber’s method and expressed as tissue culture infections dose 50 (TCID_50_) per mL (Panel assembly created with BioRender.com (2023) [52].

Replication properties of the ORFV^Δ121^conH1 were similar to the wild-type (WT) virus (OV IA82) in OFTu cells (Figure 2a). In contrast, a marked growth defect for both WT OV-IA82 and ORFV^Δ121^conH1 was observed in primary STu cells (Figure 2b), indicating minimal or no virus replication in cells of porcine origin, as previously found in other studies [37,40].

### 3.2. Recombinant ORFV^Δ121^conH1 Expresses conH1 In Vitro

**Figure 3:**
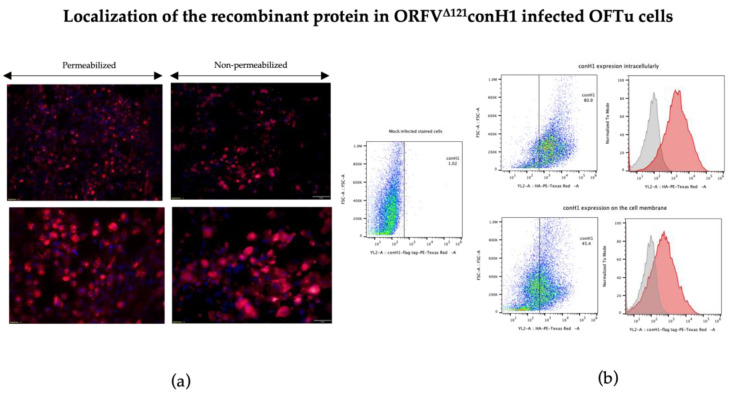
Localization of heterologous conH1 protein expressed by ORFV^Δ121^conH1. (**a**) Immunofluorescence assay in permeabilized (**left**) and non-permeabilized (**right**) OFTu cells. Expression of conH1 (red fluorescence) by the recombinant virus in intracellular compartments (**left**) as well as on the cell membrane (**right**) at 1 MOI after 48 h of infection. Blue fluorescence indicates nuclear staining by DAPI. (**b**) Expression of heterologous proteins by ORFV^Δ121^conH1 assessed by flow cytometry. OFTu cells were infected with ORFV^Δ121^conH1 or wild-type OV IA82 as a negative control. Infected cells were collected 48 h post-infection, fixed, permeabilized or not, and stained with appropriate antibodies for flow cytometric analysis. The gates indicated by rectangles gather positive stained cells (Panel assembly created with BioRender.com (2023) [52].

Expression of conH1 by ORFV^Δ12^1conH1 recombinant virus was assessed by immunofluorescence (IFA) and flow cytometry assays. Expression of conH1 by the recombinant ORFV^Δ121^conH1 was assessed during virus infection in OFTu cells by using an anti-FLAG mAb with IFA and flow cytometry assays. Importantly, these studies indicate expression of conH1 intracellularly, as showed by the intense fluorescence in permeabilized cells, and on the surface of infected cells, as shown by immunofluorescence staining in non-permeabilized ORFV^Δ121^conH1-infected cells (Figure 3a,b). Additionally, IFA assays performed in infected cells performed with serially passaged ORFV^Δ121^conH1 showed stable expression of the full-length of the inserted conH1 by the recombinant virus on passages 1, 5, and 10. Together, these results demonstrate robust expression of conH1 in cells infected with the recombinant ORFV^Δ121^conH1 virus.

### 3.3. Cross-Reactivity between H1N1 Porcine Antisera and the conH1 In Vitro

We further investigated if antibodies present in porcine antisera induced by infection with divergent H1N1 strains were able to recognize B cell epitopes in the conH1. Three different antisera from H1N1 strains isolated between 2005–2009 representing different IAV-S clades that circulate in the U.S. were used in this assay: anti-A/California/04/2009 (H1N1) belonging to the pandemic clade, -A/swine/IL/00685/2005 (H1N1) from clade delta2, and -A/swine/Kentucky/02086/2008 (H1N1) from clade beta. Cross-reactivity and antibody binding to the conH1 was observed with all three antisera, suggesting that the conH1 expressed by the recombinant ORFV^Δ121^conH1 virus was recognized by antibodies present in the sera of swine infected by divergent H1N1 viruses (Supplementary Figure S1a). Alignment of the conH1 amino acid sequences with the HA proteins of these cross-reactive viruses was performed to evaluate the genetic relationship between these viruses (Supplementary Figure S1b,c). The conH1 sequence used to construct ORFV^Δ121^conH1 possessed the lowest amino acid difference (12.4%) with the HA sequence of the virus A/swine/IL/00685/2005 (H1N1). However, higher cross-reactivity was found using the A/California/04/2009 (86.2% pairwise identity) and A/swine/Kentucky/02086/2008 (84.2% pairwise identity) sera.

### 3.4. ORFV^Δ121^conH1 Recombinant Virus Elicited Humoral Immune Response in Pigs

**Figure 4:**
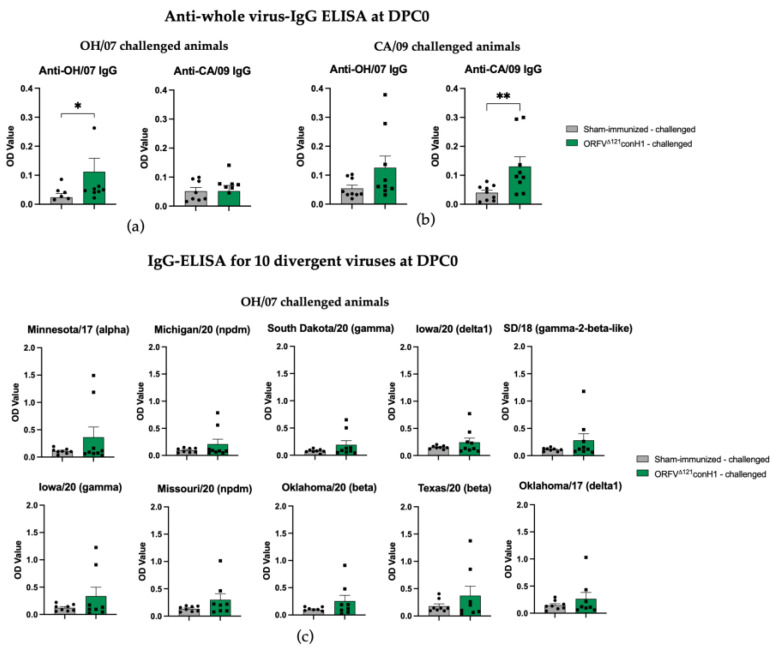

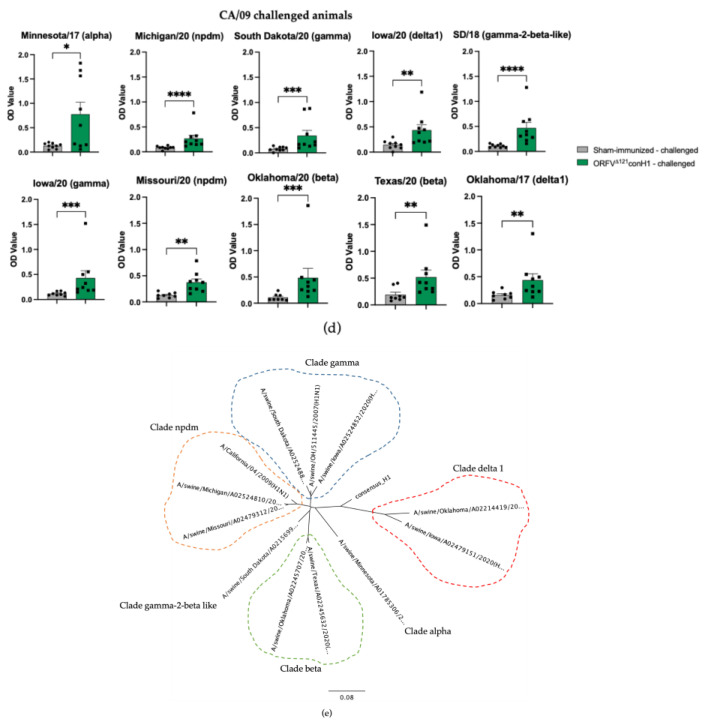
Humoral response to immunization. (**a**) IAV-S IgG responses induced by ORFV^Δ121^conH1 in OH/07- and (**b**) CA/09-challenged pigs at DPC0 (before challenge) were assessed by whole-virus ELISA using the viruses used for challenge (OH/07 and CA/09) as coating antigens. The breadth of IAV-S IgG immune response induced by ORFV^Δ121^conH1 at DPC0 was also explored against a panel of 10 divergent viruses by whole-virus ELISA for the OH/07- (**c**,**d**) and CA/09-challenged groups, respectively. (**e**) Phylogenetic tree demonstrating the distance between the HA of the viruses used for the ELISA assays and the designed consensus (consensus_H1). Each sample was tested in duplicate wells. Asterisks refer to the statistical significance between two animal groups. *p*-values: * *p* < 0.05, ** *p* < 0.01, *** *p* < 0.001, **** *p* < 0.0001.

At DPC0, IAV-S-specific total IgG antibodies were significantly higher in the immunized animals when the the challenge virus antigen was used as antigen in the ELISA (Figure 4a,b). The breadth of IAV-S IgG immune response induced by ORFV^Δ121^conH1 at DPC0 was also explored against a panel of 10 divergent viruses by whole-virus ELISA (Table 1). At DPC0, within the group of animals that were further challenged with OH/07, the immunized group showed a significant increase in total IgG levels in serum using the A/Swine/South Dakota/A02524887/2020 whole-virus as an antigen. Similar to OH/07, the A/Swine/South Dakota/A02524887/2020 virus also belongs to the U.S. H1N1 clade gamma, referred to as classical swine lineage worldwide. Notably, the nucleotide identity between the HA of conH1 used to generate the recombinant virus and the HA of A/Swine/South Dakota/A02524887/2020 is 88.1%, and in the amino acid level, the similarity was 89.1% (Table 1). Within the panel of viruses used for the whole-virus ELISA, these numbers show that the HA of A/Swine/South Dakota/A02524887/2020 shares the second highest homology with the HA of conH1, after only OH/07, with a pairwise identity of 89.2% and 89.8%, respectively (Table 1). On the other hand, within the animals challenged with CA/09, the vaccinated group showed significantly increased total IgG levels on DPC0 against A/Swine/South Dakota/A02524887/2020 (clade gamma), A/Swine/Michigan/A02524810/2020 (clade npdm), A/Swine/Texas/A02245632/2020 (beta clade), A/Swine/Oklahoma/A02245707/2020 (clade beta), and A/Swine/Oklahoma/A02214419/2017 (clade delta 1). Interestingly, these five viruses belong to different H1N1 clades, with amino acid homology ranging between 83 and 89% (Table 1). At DPC6, no difference was found between unvaccinated and vaccinated samples using the challenge viruses as antigens. Within the groups challenged with OH/07 (clade gamma), the vaccinated group maintained the significant increase in total IgG levels using A/Swine/South Dakota/A02524887/2020 (clade gamma) whole-virus as an antigen. In addition, increased levels of total IgG antibodies were detected against A/Swine/Michigan/A02524810/2020 (clade npdm), for which the HA sequence has 84.3 and 85.3% identity for nucleotides and amino acids, respectively (Table 1). Furthermore, within the groups challenged with CA/09 (clade npdm), the vaccinated group presented increased IgG cross-reactivity with A/Swine/South Dakota/A02524887/2020 (clade gamma), A/Swine/Michigan/A02524810/2020 (clade npdm), A/Swine/Oklahoma/A02245707/2020 (clade beta), and A/Swine/Oklahoma/A02214419/2017 (clade delta 1) whole-virus antigens.

**Figure 5:**
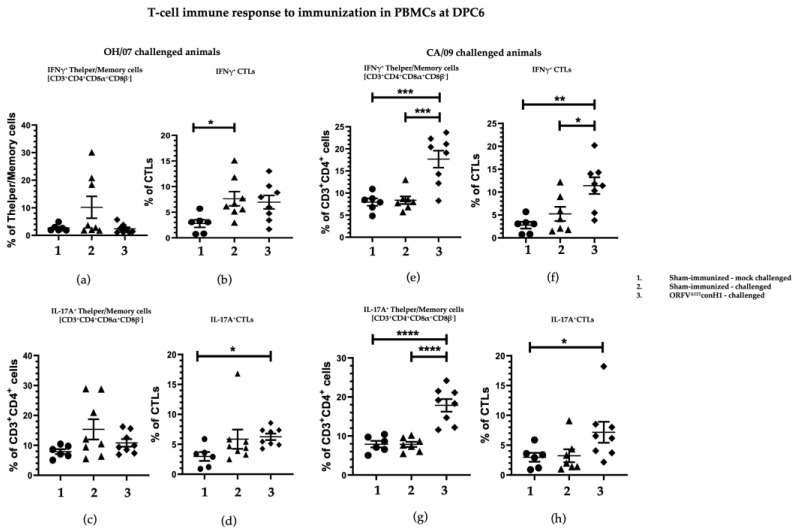
T cell response in PBMCs was evaluated by FACS. PBMCs isolated from pigs at DPC6 following recall stimulation with 0.1 MOI of OH/07 were analyzed for (**a**) IFNγ+ T helper/memory cells, (**b**) IFNγ+ CTLs cells, (**c**) percentage of IL-17A+ CTLs, and (**d**) IFNγ+ CTLs cells. PBMCs isolated from pigs at DPC6 following recall stimulation with 0.1 MOI of CA/09 were also analyzed for the same T cell populations, respectively (**e**–**h**). Asterisks refer to the statistical significance between two animal groups. *p*-values: * *p* < 0.05, ** *p* < 0.01, *** *p* < 0.001, **** *p* < 0.0001.

ORFV^Δ121^conH1 recombinant virus induced cellular immune response in pigs. T cell responses elicited by immunization with ORFV^Δ121^conH1 virus were assessed on peripheral blood mononuclear cells (PBMCs) and mononuclear cells from bronchoalveolar lavage (BAL) and tracheobronchial lymph nodes (TBLN) at DPC6. The percentage of T helper/memory cells (CD3+CD4+CD8α+β-) secreting IFN-gamma (IFNγ+) (Figure 5a,e) and IL-17 (IL17+) (Figure 5g) in PBMCs was significantly higher in the vaccinated groups. Similarly, the frequency of IFNγ+ cytotoxic T lymphocytes (CTLs) was also increased in ORFV^Δ121^conH1-immunized groups compared to unvaccinated animals challenged with CA/09 (Figure 5g). Differences in percentage of IL17+ CTLs were only observed between immunized animals and the respective sham-immunized/mock-challenged group (Figure 5d,h).

**Figure 6:**
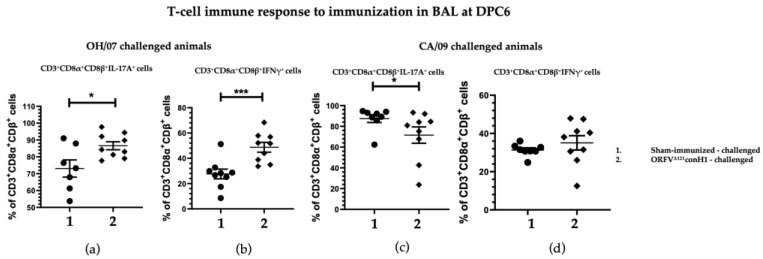
T cell immune response to immunization. BAL MNCs cells isolated from pigs at DPC6 following recall stimulation with 0.1 MOI of OH/07 were analyzed for (**a**) percentage of IL-17A+ T lymphocytes and (**b**) IFNγ+ T lymphocytes (right). BAL MNCs isolated from pigs at DPC6 following recall stimulation with 0.1 MOI of CA/09 were analyzed for the same T cell populations (**c**,**d**). Asterisks refer to the statistical significance between two animal groups. *p*-values: * *p* < 0.05, *** *p* < 0.001.

In BAL, the percentage of T cells CD3+CD8α+CD8β+ secreting IL17A and IFN-γ induced upon stimulation were significant higher in the vaccinated compared to unvaccinated animals in the groups challenged with OH/07 at DPC6 (Figure 6a,b), but no difference was found for IFN-γ secreting cells in the groups that received the CA/09 challenge (Figure 6c,d).

**Figure 7:**
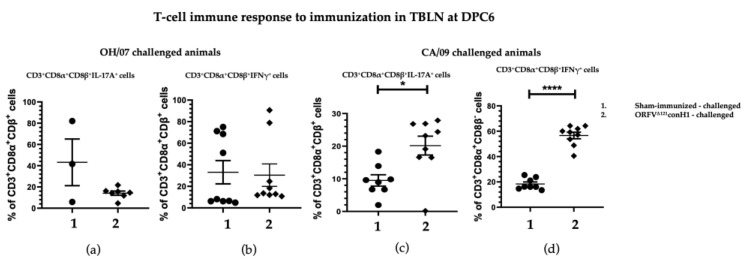
T cell immune response to immunization. TBLN MNCs cells isolated from pigs at DPC6 following recall stimulation with 0.1 MOI of OH/07 were analyzed for: (**a**) Percentage of IL-17A+ T lymphocytes and (**b**) IFNγ+ T lymphocytes (**right**). TBLN MNCs isolated from pigs at DPC6 following recall stimulation with 0.1 MOI of CA/09 were analyzed for the same T cell populations (**c**,**d**). Asterisks refer to the statistical significance between two animal groups. *p*-values: * *p* < 0.05, **** *p* < 0.0001.

In comparison, the levels of IL-17A+ and IFNγ+ secreting T cells CD3+CD8α+CD8β+ in TBLN MNC of swine vaccinated and challenged with CA/09 were significantly higher at DPC6 compared to sham-immunized pigs (Figure 7c,d), whereas no difference was found in the OH/07-challenged groups (Figure 7a,b).

**Figure 8:**
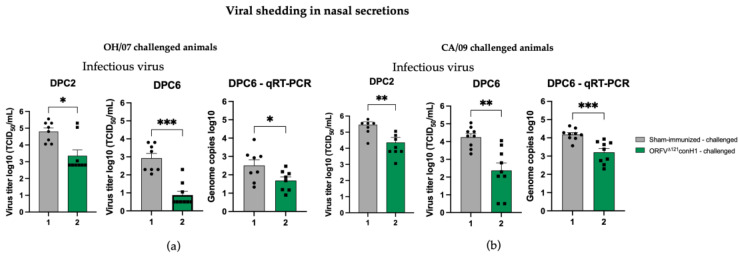
Viral load in nasal swabs were evaluated by infectious virus titration and qRT-PCR. (**a**) Viral shedding was determined in OH/07-challenged pigs by infectious titer (**left**) and (**right**) genome copy numbers. Similarly, viral shedding was also assessed in CA/09-challenged pigs (**b**) by infectious titer (**left**) and (**right**) genome copy numbers. Asterisks refer to the statistical significance between two animal groups. *P*-values: * *p* < 0.05, ** *p* < 0.01, *** *p* < 0.001.

The protective efficacy of ORFV^Δ121^conH1 was evaluated upon challenge with OH/07 and CA/09, by assessing virus shedding following intranasal challenge. The 10-fold decrease in the infectious virus present in the nasal swabs of vaccinated groups compared to their respective sham-immunized group at DPC2 (Figure 8a,b) suggests early control of virus replication in immunized animals. A significant decrease (approximately 20-fold) in infectious virus and genome copy numbers was also observed at DPC6 in nasal swabs of vaccinated groups compared to their respective sham-immunized group (Figure 8a,b). These results demonstrate that immunization with ORFV^Δ121^conH1 resulted in decreased shedding of the infectious virus and lower genome viral loads in nasal secretions following IAV-S challenge with diverse H1N1 challenge viruses.

**Figure 9:**
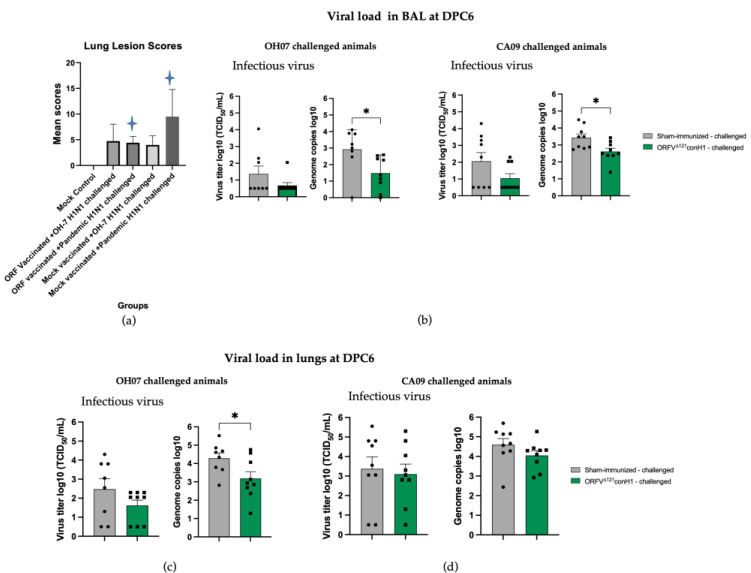
Lung lesions scores, viral infectious titer, and viral load were assessed in BAL and lung lysates of sham-immunized/challenged and ORFV^Δ121^conH1-challenged pigs on DPC6. (**a**) Lung scores were assessed on DPC6. Blue stars refer to the statistical significance between two animal groups, where *p* < 0.05, (**b**) IAV-S viral RNA shedding in the BAL of swine was determined by TCID_50_ mL^−1^ (**left**) and RT-qPCR (**right**). (**c**) Viral infectious titer (**left**) and viral load (**right**) in the lungs of swine sham- and ORFV^Δ121^conH1-immunized pigs challenged with either OH/07 or (**d**) CA/09 on DPC6. Asterisks refer to the statistical significance between two animal groups. *p*-values: * *p* < 0.05.

Macroscopic lesions in the lung were also evaluated. The lesion scores were significantly lower in ORFV^Δ121^conH1-immnunized group when compared to sham-immunized animals that were challenged with the same CA/09 virus (Figure 9a). Finally, viral load was determined in the BAL of sham- and ORFV^Δ121^conH1-immunized pigs at DPC6 by genome copies mL^−1^ (log 10) and TCID_50_ mL^−1^. Despite the 10-fold lower load of IAV-S RNA detected in the BAL of vaccinated animals, there was no significant difference in terms of infectious virus titers between sham- and ORFV^Δ121^conH1-immunized groups (Figure 9b). Taken together, these results demonstrate that immunization with ORFV^Δ121^conH1 was able to reduce viral shedding after intranasal infection with divergent strains of IAV-S H1N1 (OH/07 and CA/09) and diminish lung lesions upon infection with a pandemic virus isolate (CA/09).

## 4. Discussion

Overcoming the outcome of wide genetic diversity of IAV-S is challenging. The in silico design of a consensus sequence of the immunogenic viral protein based on a large number of circulating virus sequences can reduce the average genetic and antigenic distances between the heterologous gene/protein and circulating wild-type viruses. This approach leads to the generation of antibodies and T cell responses that react to a broader range of viral strains. Previously, a consensus-based human immunodeficiency virus type 1 (HIV- 1) vaccine reduced the average genetic distance of the consensus to field strains by half [53]. Later, consensus-based HIV-1 immunogens were demonstrated to induce a broader level of protection than naturally occurring antigens [54,55]. In the case of influenza A viruses, since HA is the most abundant envelope protein and highly immunogenic, studies have been conducted to assess the protective immunity conferred by a consensus of HA for human and avian IAVs [24,56,57,58,59,60,61,62,63]. Recently, a consensus vaccine based on the HA of H3 viral subtype elicited broader levels of protective immunity in pigs against IAV-S than the HA of the field virus H3N2 IAV-S strain TX98 [60]. Here, we showed that a consensus designed based on the HA of the highly diverse H1 subtype of IAV-S reduced the pairwise genetic difference from field IAV-S isolates and resulted in partial protection against diverse IAV-S strains of the gamma and npdm clades.

The lack of neutralizing antibodies found within the conH1-vaccinated pigs might be caused by the consensus sequence design, and/or by the vaccine regimen and dose. A study using a new generation of computationally optimized broadly reactive antigens (COBRA)-based vaccines demonstrated that the isotype of elicited Ab was influenced by the vaccination regimen. Mice immunized through an intranasal route followed by an intraperitoneal immunization showed higher levels of IgG HA Abs, while the animals primed and boosted intramuscularly presented the lowest titers for all IgG subclasses [64]. Considering that hemagglutination inhibition (HI) titers equal or greater than 40 are associated with protection against influenza in adult human subjects, a study showed that mice vaccinated with adenoviral vectors expressing a consensus of H1 demonstrated that HI titers raised in a dose response manner [56,62]. Here, we did not perform a dose and regimen optimization of the vaccine candidate, which might explain the suboptimal levels of neutralizing antibodies (nAbs). Indeed, consensus immunogens generally lie in cellular immunity to protect against disease; nevertheless, cross-reactive non-neutralizing antibodies able to induce antibody-dependent cellular cytotoxic (ADCC) may be beneficial. Similar to our findings, a consensus-based DNA vaccine for avian influenza detected high levels of anti-HA antibody by ELISA in immunized mice sera; however, they lacked neutralizing activity [63]. These results extend to consensus-based vaccine candidate for other viruses as well. Previously, the same research group showed that a human immunodeficiency virus (HIV) subtype B consensus-based vaccine using an envelope gene (EY2E1-B) demonstrated potent cross-reactive cellular immune responses, but no antibody responses against divergent subtypes of HIV [64]. In order to enhance cross-reactive nAb responses, a sequential immunization with vaccines based in the resulting consensus of HA from different subtypes could redirect the immune responses towards conserved epitopes of the HA glycoprotein, as shown by Zhou et al. (2017) [59].

T cell responses are critical against influenza virus infection and are especially important for virus clearance for broadening the breadth of protection [32,65,66,67,68]. ORFV^Δ121^conH1-immunized/CA/09-challenged pigs had a significant higher frequency of IFNγ+ CTLs in PBMCs and TBLN-MNC, indicating the induction of influenza-specific CD8+T cells (Table S2). Moreover, the frequency of IL-17A+ and IFNγ+CD8+ T lymphocytes in TBLN-MNC were significantly higher at DPC6 than those of sham-immunized/CA/09-challenged pigs, suggesting induction of influenza-specific CD8+ T cells. In addition to their direct role in viral clearance, CD8+ T cells, have a role in mediating heterosubtypic immunity against influenza viruses [30,68,69].

Similar to the results observed for CTLs, the frequencies of IL-17A+ and IFNγ+CD4+ T helper/memory cells were increased substantially at DPC6 in ORFV^Δ121^conH1-immunized/CA/09-challenged pigs compared to unvaccinated animals subjected to the same challenge virus. Importantly, influenza-specific CD4+ T cells have been correlated with protection against disease even in humans lacking cross-protective antibodies. The CD4+ T cell population stimulates humoral responses and virus specific CTLs, which may have contributed to the overall protection observed in ORFV^Δ121^conH1-immunized animals [67]. Memory T cells and conserved epitopes have been implicated in protection to heterosubtypic infection [68,70]. In ferrets lacking sterilizing immunity after prior exposure to a seasonal H1N1 influenza, there was protection from disease upon subsequent infection with a H1N1 belonging to a different clade [71]. In humans, lower hospitalization and infection reports was observed during the H1N1 pandemic among people recently vaccinated against seasonal influenza in Mexico in 2009 [72]. Other studies also showed correlation between cross-reactive T cell-specific responses for internal IAV proteins and protection against symptomatic disease [73,74].

It has been observed that IAV-S OH/07 replicates very efficiently in the lungs, causing extensive lesions, and can be detected at higher titers in the lungs but not in other tissues [15]. In contrast, a study involving pigs infected with the CA/09 strain revealed pathological changes in both respiratory and lymphoid tissues, and at 7 days post-challenge, viral RNA was still detected in local lymph nodes by PCR [75]. This observation could be linked to the increased cellular-mediated immunity (CMI) seen in the BAL of vaccinated pigs challenged with OH/07, while no significant difference was observed in the TBLN. The differences in the pathogenesis and/or replication ability of these strains may account for some of the variations in cellular-mediated immunity. In a comparative study examining various H1N1 IAV-S strains, CA/09-inoculated pigs exhibited higher viral loads in their nasal cavities compared to two other strains at 5 dpi [76]. In our study, we found that the CA/09-challenged pigs in both the vaccinated and unvaccinated groups had higher viral titers than the pigs that received OH/07. These differences could be related to inherent differences in tropism and replication of OH/07 and CA/09 or yet on their ability to modulate host innate and adaptive immune responses in vivo.

T cell mediated protection can also be mediated by tissue-resident memory T cells (TRMs), which reside at the tissue of the primary infection, such as the lungs, in the case of IAV [77,78]. Although TRMs cannot provide sterilizing immunity, they can prevent aggravation of disease and lung pathology by restricting virus replication. Importantly, mice studies demonstrated the role of lung CD8+ and CD4+ TRMs against heterologous IAV infections [79,80]. Here, we found higher percentage of T cells in the vaccinated groups challenged with either OH/07 or CA/09 in BAL or local lung draining lymph nodes (TBLN), respectively. These observations might be suggestive of activation of TRMs, but further studies are needed to assess the phenotypic profile of these T cells by using specific markers, such as the C-type lectin CD69 and the integrin CD103 [78,79].

ORFV has emerged as a promising delivery vector for foreign antigens in animals due to its narrow natural host range (sheep and goats), the non-systemic self-limiting infection, the lack of neutralizing antibodies against the vector which enables booster immunizations, and because of its natural immunogenicity, inducing long-lasting humoral and T cell responses against heterologous antigens [81,82]. Additionally, the defective replication of ORFV in swine cells is another important feature that adds to its safety profile. We have successfully delivered several antigens using the ORFV vector in livestock species, including the full length of a codon-optimized HA derived from an OH/07 strain [37]. In this study, the ORFV-based constructs comprising exclusively the HA or both HA and NP proteins elicited high levels of neutralizing antibodies and T cell responses, and decreased infectious virus shedding in nasal secretions, protecting pigs against a homologous challenge. Here, cross-reactive antibodies and protective T cell responses were elicited by the ORFV vector encoding the conH1 sequence, suggesting that this strategy could be useful in providing broader protection against divergent IAV-S. Interestingly, in Joshi et al. (2021), the recombinant ORFV virus expressing both HA and NP was found to induce a greater immunogenicity and protective efficacy, while an NP-expressing ORFV recombinant failed to protect mice against H5N1 lethal challenge [83]. These findings suggest the conserved NP by itself is not enough to induce strong immune responses against influenza, but the inclusion of NP can contribute to protection through the activation of specific CTLs mediated by conserved epitopes, accelerating viral clearance [84,85,86,87]. Future studies involving the inclusion of NP to the ORFV-conH1 can aid in determining if the addition of this conserved domain could improve the immune responses and protection against IAV-S infections in swine.

In summary, the recombinant ORFV expressing a consensus of HA for H1 subtype induced T helper/memory cells and CTLs in the pig lungs against CA/09, a strain belonging to the H1N1 pandemic clade. Furthermore, the increased levels of T lymphocytes in the BAL suggest local mucosal protection against OH/07, a strain from the H1N1 gamma clade. Further studies are needed to evaluate the cross-reactivity of these responses, and the breadth of protection against a range of heterologous viruses driven by consensus- based swine influenza vaccines. Based on our results, we believe that the delivery of a consensus sequence of the highly immunogenic hemagglutinin of IAV-S is a useful strategy to design novel recombinant vaccines that will be able to protect against diverse IAV-S strains. Given the constant evolution of IAV-S, it is likely that the consensus sequence may need to be updated from time to time to increase the breath of protection against contemporary strains.

## Figures and Tables

**Table 1:** Information on the IAV-S isolates used for the ELISAs, including the percent nucleotide (nt) and amino acid (aa) pairwise identities to the conH1 sequence.

Info	Ohio/07	California/09	Iowa/20	Iowa/20	Michigan/20	Minnesota/17	Missouri/20	Oklahoma/17	Oklahoma/20	South Dakota/18	South Dakota/20	Texas/20
Clade	gamma	Npdm	gamma	delta 1	npdm	alpha	npdm	delta 1	beta	gamma-2-beta-like	gamma	beta
Year	2007	2009	2020	2020	2020	2017	2020	2017	2020	2018	2020	2020
State	Ohio	California	Iowa	Iowa	Michigan	Minnesota	Missouri	Oklahoma	Oklahoma	South Dakota	South Dakota	Texas
Isolate number	511445	4	A02524852	A02479151	A02524810	A01785306	A02479312	A02214419	A02245707	A02156993	A02524887	A02245632
Nt pairwise id conH1 (%)	89.2	86.1	88	85	84.3	82.5	84.6	83.6	83.7	86.9	88.1	83.7
Aa pairwise id conH1 (%)	89.8	85.9	88.3	85.2	85.3	83.9	85.5	82.9	83.4	86.9	89.1	83.7

**Table 2:** Experimental design of animal immunization-challenge study.

Group	*n*	Immunization	Immunogen Titer	Route	Immunization Days	Challenge	Inoculum Titer	Route	Challenge Day
1	6	Sham	–	Intramuscular	0, 21 DPV *	Mock (MEM)	–	Intranasal and intratracheal	40 DPV
2	9	ORFV^Δ121^conH1	2 × 10^7.5^ mL^−1^	Intramuscular	0, 21 DPV	OH/07 (gamma)	1 × 10^7.0^/route	Intranasal and intratracheal	40 DPV
3	9	ORFV_Δ121_conH1	2 × 10^7.5^ mL^−1^	Intramuscular	0, 21 DPV	CA/09 (npdm)	1 × 10^7.0^/route	Intranasal and intratracheal	40 DPV
4	9	Sham	–	Intramuscular	0, 21 DPV	CA/09 (npdm)	1 × 10^7.0^/route	Intranasal and intratracheal	40 DPV
5	8	Sham	–	Intramuscular	0, 21 DPV	OH/07 (gamma)	1 × 10^7.0^/route	Intranasal and intratracheal	40 DPV

* DPV = days post-vaccination.

## Data Availability

All data is presented within the manuscript or as supporting information.

## Supplementary Materials

The following supporting information can be downloaded at: https://www.mdpi.com/article/10.3390/v15040994/s1, Figure S1: Cross-reactivity between divergent porcine antisera and ORFV^Δ121^conH1. Table S1: Set of the 63 HA sequences used for the nucleotide alignment that resulted in the conH1 sequence; Table S2: Summary of significantly upregulated immune cells.
