# Supplementary material for: An Orf-Virus (ORFV)-Based Vector Expressing a Consensus H1 Hemagglutinin Provides Protection against Diverse Swine Influenza Viruses
Source: Viruses. 2023 Apr 18;15(4):994. doi: 10.3390/v15040994 (PMC10147081; doi:10.3390/v15040994)
Supplement: Supplementary file 1 [file viruses-15-00994-s001.zip › viruses-2308473-supplementary.pdf]

| Access number | Name | Submission date |
| --- | --- | --- |
| **KJ683871** | A/swine/South Dakota/A01480750/2014 | 3/27/14 |
| **KY802128** | A/swine/NorthCarolina/A01672751/2017 | 2/8/17 |
| **MF692781** | A/swine/North Carolina/A01785281/2017 | 7/28/17 |
| **MF150370** | A/swine/North Carolina/A02214775/2017 | 4/19/17 |
| **KY014451** | A/swine/North Carolina/A01781336/2016 | 9/14/16 |
| **KY499597** | A/swine Oklahoma/A01671948/2016 | 12/22/16 |
| **KX772389** | A/swine/Iowa/A01778178/2016 | 8/3/16 |
| **KU379628** | A/swine/Oklahoma/A01798209/2015 | 11/23/15 |
| **MF455481** | A/swine/Iowa/A02216456/2017 | 5/23/17 |
| **MF359749** | A/swine/Iowa/A01785264/2017 | 5/30/17 |
| **KX380247** | A/swine/Illinois/A01775513/2016 | 5/19/16 |
| **KX185834** | A/swine/Illinois/A01895276/2016 | 4/8/16 |
| **MF000481** | A/swine/Missouri/A01672817/2017 | 2/6/17 |
| **KR265520** | A/swine/Missouri/A01554380/2015 | 4/24/15 |
| **MF144718** | A/swine/Iowa/A02215041/2017 | 5/19/17 |
| **MF348036** | A/swine/North Carolina/A02215469/2017 | 5/17/17 |
| **KY744620** | A/swine/Indiana/A01672825/2017 | 2/1/17 |
| **MG383438** | A/swine/Iowa/A01678511/2017 | 1/11/17 |
| **KY995615** | A/swine/Nebraska/A02214231/2017 | 3/22/17 |
| **MF289445** | A/swine/Iowa/A02215255/2017 | 5/8/17 |
| **KY744614** | A/swine/Iowa/A01672823/2017 | 2/2/17 |
| **MF348031** | A/swine/Missouri/A02216048/2017 | 5/16/17 |
| **KX298117** | A/swine/North Carolina/A01774921/2016 | 5/9/16 |
| **KU942633** | A/swine/Indiana/A01732606/2016 | 2/29/16 |
| **KU877392** | A/swine/North Carolina/A01731797/2016 | 2/17/16 |
| **KX518666** | A/swine/North Carolina/A01776155/2016 | 5/31/16 |
| **KU942615** | A/swine/North Carolina/A01732321/2016 | 2/22/16 |
| **KR863122** | A/swine/Illinois/A01820485/2015 | 5/22/15 |
| **KU695675** | A/swine/North Carolina/A01730376/2016 | 1/22/16 |

| | | |
|---|---|---|
| **EU604689** | A/swine/OH/511445/2007 | 4/1/08 |
| **KU680986** | A/swine/Iowa/A01941927/2016 | 1/14/16 |
| **MF471674** | A/swine/Iowa/A02217286/2017 | 6/15/17 |
| **MF613966** | A/swine/Iowa/A02218176/2017 | 6/28/17 |
| **MF582488** | A/swine/Nebraska/A01785277/2017 | 7/14/17 |
| **KX150858** | A/swine/Oklahoma/A01676763/2016(H1N2) | 4/27/16 |
| **MF455467** | A/swine/Indiana/A02217290/2017(H1N2) | 7/10/17 |
| **KY766094** | A/swine/Minnesota/A01932042/2017(H1N2) | 3/13/17 |
| **KY522887** | A/swine/Iowa/A01672046/2017 | 1/5/17 |
| **MF375251** | A/swine/Iowa/A02216454/2017 | 5/22/17 |
| **MF664424** | A/swine/Oklahoma/A01785279/2017 | 7/18/17 |
| **KY941155** | A/swine/Missouri/A01667101/2017 | 3/8/17 |
| **MF092725** | A/swine/Michigan/A02214665/2017 | 3/28/17 |
| **MF092737** | A/swine/Iowa/A02214655/2017(H1N2) | 5/11/17 |
| **KM402870** | A/swine/South Dakota/A01482581/2014(H1N2) | 8/27/14 |
| **KY631495** | A/swine/Iowa/A01672519/2017 | 1/20/17 |
| **KY412996** | A/swine/Iowa/A01668923/2016 | 11/30/16 |
| **MF455505** | A/swine/Iowa/A02216639/2017 | 6/7/17 |
| **MF488955** | A/swine/South Dakota/A01678497/2017(H1N2) | 7/14/17 |
| **KX602673** | A/swine/Minnesota/A01678467/2016(H1N2) | 7/25/16 |
| **MF150384** | A/swine/Illinois/A02214842/2017 | 4/10/17 |
| **KY995593** | A/swine/Indiana/A01667098/2017 | 3/3/17 |
| **KY210992** | A/swine/North Carolina/A01782869/2016 | 10/27/16 |
| **MF455485** | A/swine/Iowa/A02216640/2017 | 6/6/17 |
| **MF092761** | A/swine/North Carolina/A02214480/2017 | 4/7/17 |
| **KY888291** | A/swine/North Carolina/A01932634/2017 | 3/13/17 |
| **MF768424** | A/swine/Pennsylvania/A02218034/2017 | 7/5/17 |
| **MF768426** | A/swine/Iowa/A02217969/2017 | 6/28/17 |
| **KU877362** | A/swine/North Carolina/A01731857/2016 | 2/11/16 |
| **KY744610** | A/swine/North Carolina/A01672678/2017 | 2/3/17 |
| **KY593209** | A/swine/North Carolina/A01672380/2017 | 1/19/17 |

| | | |
|---|---|---|
| **KY465593** | A/swine/Pennsylvania/A01671487/2016 | 12/12/16 |
| **KY873258** | A/swine/North Carolina/A01932425/2017 | 2/16/17 |
| **KY631499** | A/swine/North Carolina/A01672458/2017 | 1/20/17 |

ConH1 sequence:
ATGAAGGCAATACTATTAGTTCTACTATATACATCTACAACTGCAAATGCCGACAAACTATGTAT
AGGTTATCATGCGAACAACTCAACTGACACTGTAGACACAGTACTAGAAAAGAATGTAACAGTA
ACACATTCTGTTAATCTTCTAGAAAACAAGCATAATGGGAAACTATGTAAACTAAGAGGGGTAG
CTCCATTGCACTTGGGTAAATGTAACATTGCTGGCTGGCTTCTGGGAAATCCAGAGTGTGACTCA
CTCTCTACAGCAATCTCATGGTCTTACATTGTAGAAACATCTAATTCAGACAATGGAACGTGTTA
CCCAGGAGATTTCAATAATTATGAGGAGCTAAGGGAGCAGTTGAGCTCAGTGTCATCATTCGAA
AGGTTTGAGATATTCCCCAAGACAAGCTCATGGCCCAACCATGACACAGACAAAGGTGTGACGT
CAGCATGTCAACATGCTGGGAGAGGAAGCTTCTACAGAAATTTGTTATGGCTGGTCAAAAAAGA
AAATTCATATCCAAAGATCAACAAATCCTACACTAACACTAGAGGGAAAGAAGTTCTAGTGCTA
TGGGCCATTCACCACCCGCCTACCAGTGCCGACCAACAAAGTCTATACCAAAATGCAAATGCCT
ATGTTTTTGTGGGGTCATCAAAATACAGCAGGAAGTTCGAGCCAGAAATAGCAACAAGACCCAA
AGTGAGGAACCAAACAGGGAGAATGAACTATTACTGGACACTAGTAGAGCCTGGAGACAATAT
AACATTCGAAGCAACTGGAAATCTGGTGGTACCGAGATATGCCTTCGCATTGAAAAGAAATTCT
GGATCCGGTATTATCATTTCAGATACATCAGTCCACGATTGTGATACGACTTGTCAGACACCCAA
TGGTGCTATAAACACCAGCCTCCCATTTCAAAACATACACCCAGTCACAATTGGAGAATGTCCA
AAATATGTAAAAAGTACTAAACTGAGAATGGCCACAGGATTAAGGAATATCCCTTCCATTCAAT
CTAGAGGCCTGTTTGGGGCAATTGCCGGCTTTATTGAAGGAGGCTGGACAGGAATGATAGATGG
GTGGTACGGTTATCATCATCAAAATGAGCAGGGATCAGGGTATGCAGCCGACCTGAAGAGCACA
CAGAGTGCCATTGACGGAATCACTAACAAGGTTAATTCTGTTATTGAAAAGATGAACACACAAT
TCACTGCTGTAGGTAAAGAGTTCAGCCACTTGGAAAGGAGAATAGAGAATTTAAACAAAAAGGT
TGATGATGGGTTTCTGGACATTTGGACTTACAATGCCGAACTGTTGGTTCTATTGGAAAATGAAA
GAACTCTGGATTACCACGATTCAAATGTGAAAACCTTGTATGAAAAGGTAAGAAGCCAACTAAA
AAACAATGCCAAGGAAATTGGAAATGGCTGTTTTGAATTTTACCACAAATGTGATGACACGTGC
ATGGAAAGCGTCAAAAATGGAACTTATGACTACCCAAAGTACTCAGAGGAAGCAAAACTAAAC
AGGGAGGAAATAGATGGGGTAAAGTTGGAATCAACAAGGATTTACCAAATTTTGGCGATCTATT
CAACAGTCGCCAGTTCGTTGGTACTGGTAGTCTCCCTGGGGGCAATCAGTTTCTGGATGTGCTCTA
ATGGGTCGCTACAGTGCAGAATATGTATTTAA

**Table S1.** Set of the 63 HA sequences used for the nucleotide alignment that resulted in the conH1 sequence listed above.

**Cross-reactivity between H1N1 porcine antisera and ORFV$^{\Delta121}$conH1**

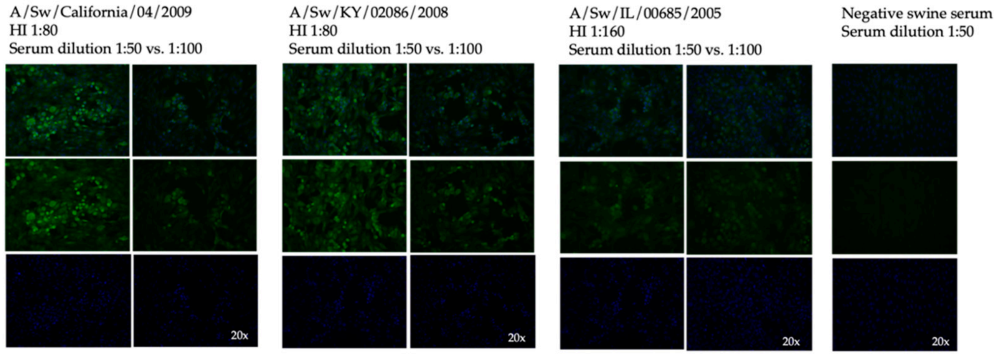


(a)

| Info | A/Sw/California/04/2009 | A/Sw/KY/02086/2008 | A/Sw/IL/00685/2005 |
|---|---|---|---|
| clade | npdm | beta | delta 2 |
| year | 2009 | 2008 | 2005 |
| state | California | Kentucky | Illinois |
| isolate number | 4 | 2086 | 685 |
| Nt pairwise id conH1 (%) | 86.2 | 84.2 | 86.2 |
| Aa pairwise id conH1 (%) | 86.2 | 83.7 | 87.6 |

(b)

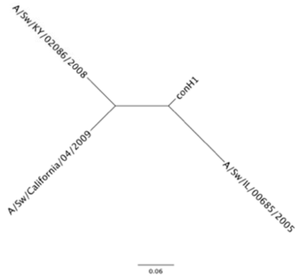


(c)

**Figure S1.** Cross-reactivity between divergent porcine antisera and ORFV$^{\Delta121}$conH1**.** (a) The cross-reactivity between serum of pigs infected with 3 different strains of H1N1 IAV-S and conH1 expressed on the cell surface of OFTu permeabilized cells was assessed by IFA. Positive cross-reactivity is showed by the green fluorescence, suggesting that the conH1 expressed by the recombinant ORFV$^{\Delta121}$conH1 virus was recognized by the host serum from infected animals. (b) Informative table about the IAV-S isolates used for the cross-reactivity sera assay, including the pairwise identities (c) Phylogenetic tree based on the nucleotide sequences of the HAs from these same isolates to evaluate genetic distance of with the conH1.

| **Immune cell phenotypes** | **Animals challenged with A/Sw/OH/24366/2007** | **Animal challenged with A/California/04/2009** |
|---|---|---|
| IL-17A$^+$ CTLs in PBMCs [CD3$^+$CD4$^+$CD8$\alpha^+$CD8$\beta^+$IL-17A$^+$] | *P < 0.05 | *P < 0.05 |
| IL-17A$^+$ T-helper/Memory cells in PBMCs [CD3$^+$CD4$^+$CD8$\alpha^+$CD8$\beta^-$IL-17A$^+$] | - | ****P < 0.0001. |
| IFN$\gamma^+$ T-helper/Memory cells in PBMCs [CD3$^+$CD4$^+$CD8$\alpha^+$CD8$\beta^-$ IFN$\gamma^+$] | *P < 0.05 | ***P < 0.001 |
| IFN$\gamma^+$CTLs in PBMCs [CD3$^+$CD4$^+$CD8$\alpha^+$CD8$\beta^+$ IFN$\gamma^+$) | *P < 0.05 | *P < 0.05, **P < 0.01 |
| IL-17A$^+$ T-lymphocytes in BAL cells [CD3$^+$CD8$\alpha^+$CD8$\beta^+$IL-17A$^+$] | *P < 0.05 | *P < 0.05 |

| | | |
|---|---|---|
| IFNγ$^+$ T-lymphocytes in BAL cells [CD3$^+$CD8α$^+$CD8β$^+$IFNγ$^+$] | ***P < 0.001 | - |
| IL-17A$^+$ T-lymphocytes in TBLN MNCs [CD3$^+$CD8α$^+$CD8β$^+$IL-17A$^+$] | - | *P < 0.05 |
| IFNγ$^+$ T-lymphocytes in TBLN MNCs [CD3$^+$CD8α$^+$CD8β$^+$IFNγ$^+$] | - | ***P < 0.001 |

**Table S2.** Summary of significantly upregulated immune cells,
